# Editorial: Expert opinion in environmental and genetic factors impacting functional brain lateralization in development and evolution

**DOI:** 10.3389/fnbeh.2023.1215176

**Published:** 2023-05-31

**Authors:** Gianluca Malatesta, Luca Tommasi

**Affiliations:** Department of Psychological, Health and Territorial Sciences, University ‘G. d'Annunzio' of Chieti-Pescara, Chieti, Italy

**Keywords:** lateralization and brain functions, social behavior, environmental factors, epigenetics, comparative & evolutionary neuroscience

Functional lateralization of the brain (i.e., the asymmetrical distribution of functions in the two structurally symmetrical cerebral hemispheres), is a research field that has progressed immensely before and after the turn of the Millennium, incorporating inputs from many aspects of biology (e.g., evolution, genetics, and neuroscience) and psychology (e.g., cognition, emotion, and their associated disorders; Ocklenburg and Gunturkun, 2017; Vingerhoets, 2019). However, a sense of stasis might appear to afflict the entire field even to the eyes of the knowledgeable observer, as many of the advancements do not seem to have really added much to the understanding of key questions, for instance in the field of human health (Ocklenburg et al., 2021). We believe that the field indeed epitomizes a most impressive case in which descriptive simplicity (i.e., the search for “simple” left vs. right differences) faces the explanatory complexity of how complex nervous systems originate from complex genes that exert their effects in complex environments. However, sometimes appearances are deceiving, and this is especially true if the simplicity of “left vs. right” is assumed at face value (Marzoli et al., 2022), forgetting that it is a result of that complexity but it might as well be one of its drivers—in evolutionary and developmental terms.

This Research Topic presents an overview of expert opinions in what we deem a righteous recognition of a domain that is by no means simple, and that is succeeding in integrating knowledge on functional lateralization. Such integration takes please by means of the study of individual and social behaviors in humans and non-human species, the computational modeling of their evolutionary constraints, the genetics and epigenetics of typical and atypical neural development—to name but a few disparate sources of evidence that well represent the liveliness of the field.

Pfeifer et al. start from circumscribing the contribution of large-scale genetic and epigenetic association studies on lateralization phenotypes. These have been traditionally oversimplified for the sake of rapid assessment (e.g., left vs. right dominant hand), not doing full justice to the understanding of how the complexity of behavioral asymmetries is linked to genetic and epigenetic causation. Because behavioral lateralization involves a much more articulate pattern, especially when looked through the lens of social interaction, their proposal to expand the laterality phenotype spectrum assessed in GWAS and EWAS studies (e.g., including self-reported preferences in kissing, hugging, cradling, etc.; Packheiser et al., 2020; Malatesta et al., 2021a) comes as a very reasonable and practical suggestion.

Berretz and Packheiser present an interesting point of view on the occurrence of atypical hemispheric asymmetries in clinical conditions with different levels of heritability and susceptibility to environmental stress starting early in life (Berretz et al., 2020). The authors trace a connection between the heterogeneity of environmental causes and the reduced association with alterations of functional brain lateralization observed in major depressive disorder, as compared to the less heterogeneous conditions. Although speculative, their insight might prove useful to differentiate between patients based on the association among symptom clusters, life-history, and brain asymmetry. This would represent a demonstration of the direct application of brain lateralization to novel diagnostic criteria in a field, psychiatry, which has been reluctant to incorporate neuroscientific evidence.

Nelson moves the discourse toward the development of behavioral asymmetries, specifically handedness. Strongly grounded in the tradition that documented how specific prenatal events trigger a series of developmental cascades that end up in establishing handedness directionality in humans (Michel and Harkins, 1986), she urges the adoption of a similar approach in the comparative study of primate handedness. Studies performed on a few primate species are reviewed and re-interpreted in this light, attributing to innate postural constraints the power of channeling visual experience toward one side, thus reinforcing the emergence of hand preference later in life.

Within a similar framework, Malatesta et al. identify the population-level leftward lateralization of maternal cradling during the first post-natal weeks (i.e., a critical period for the neurodevelopment of brain functions) as one of the earliest socio-environmental factors epigenetically canalizing neurodevelopment (Malatesta et al., 2021b). Moreover, they consider the left-cradling bias as a double-exchange platform of “monitoring and exposure”, which benefits both the mother and the infant and is presumably shaped by evolutionary and social pressures. In fact, both phylogenetic and ontogenetic factors are supposed to be involved in the emergence of this interactive side bias, which might subserve a similar function in human infants as that shown by means of light exposure in avians during incubation.

In this regard, Rogers traces a pattern of direct causal relationships between prenatal sensory experience and the establishment of asymmetrical behaviors known to depend on the ontogenesis of neural lateralization. The article revolves around the vast literature accumulated on the avian embryo, because it has proven a superlative animal model especially since the discovery of the effects of light exposure *in ovo* on chicken brain and behavior by Rogers (1982). The understanding of these environmental factors has expanded including precise tests of the effects of (or lack thereof) auditory and olfactory stimulation, and certainly represents a cornerstone of neuroethological epigenetics.

Comparative research is also under the Giljov and Karenina spotlight. The authors pinpoint that ungulates (saiga antelopes, especially) also might represent a convenient model for social laterality (intended as positional side-bias observation during social interactions; Karenina et al., 2017). For example, these animals do show overtly lateralized social behaviors—generally comparable with those of humans—and do not use forelimbs in social interactions (as is the case of humans and other primates). These factors could make easier the interpretation of this kind of positional and motor behaviors and possibly bring significant insights into the understanding of social laterality.

Another comparative study is that of Loconsole et al., who discuss the influence of asymmetrical spatial numerical association on numerical discrimination in chicks by recoding behavioral data from a previous study (Rugani et al., 2022). They speculate on the qualitatively different hemispheric contributions and specializations, as well as on the role of brain functional asymmetries for mapping numbers onto space during evolution.

Finally, Tonello and Vallortigara address the issue of the simulation models which so far have been proposed to account for population-level brain and behavioral asymmetries. According to one of these (Ghirlanda et al., 2009), the unbalanced ratio of left- and right- lateralized individuals can arise from an evolutionary stable strategy based on the balance between competitive (i.e., favoring individuals differently lateralized from the majority, who would be more able to surprise antagonists) and cooperative (i.e., favoring individuals showing the same lateralization) interactions. In this context, the authors provided a new probabilistic and evolutionary perspective by considering the population as a whole, within which the single individual can indirectly communicate with each other through the environmental change in a social way, as a form of “stigmergy” (Theraulaz and Bonabeau, 1999). Therefore, a system can automatically self-organize its own laterality balance by expressing a sort of “swarm intelligence”, but only when operating as a group.

## Author contributions

All authors listed have made a substantial, direct, and intellectual contribution to the work and approved it for publication.
